# Portable microwave sensor system for real-time detection of microplastics in seawater

**DOI:** 10.1038/s41378-026-01413-y

**Published:** 2026-08-24

**Authors:** Yuxuan Hou, Qingzhou Wang, Qilong Zhang, Jiaxu Liu, Eun-Seong Kim, Nam-Young Kim, Guanlin Li, Yuanyue Li, Xiaocui Wang, Zhao Yao

**Affiliations:** 1 Shandong Key Laboratory of Micro-nano Packaging and System Integration, College of Electronic and Information, Qingdao University, Qingdao, China; 2 Department of Electronic Engineering, Kwangwoon University, Seoul, South Korea; 3 Department of Molecular Medicine and Biopharmaceutical Sciences, Laboratory of Molecular Pathology and Cancer Genomics, Graduate School of Convergence Science and Technology, Seoul National University, Seoul, South Korea; 4 Department of Medical and Digital Engineering, College of Engineering, Hanyang University, Seoul, South Korea; 5 Konkuk University, Stem Cell and Regenerative Biotechnology, School of Advanced Biotechnology, Seoul, Republic of Korea; 6 School of Environment and Safety Engineering, Jiangsu University, Zhenjiang, China; 7 School of Environment and Geography, Qingdao University, Qingdao, China

**Keywords:** Electrical and electronic engineering, Electronic properties and materials

## Abstract

With the rapid development of the global plastic industry, microplastic pollution has become an increasingly serious environmental concern. However, standardized technologies for convenient, accurate, and real-time microplastic detection are limited. To address this challenge, a microwave resonant sensor with a complete microplastic detection system equipped with a Bluetooth module for remote real-time monitoring of microplastic concentrations was developed. Microplastic concentrations were measured in deionized water containing polyvinyl chloride powder (particle size: 6.5 ± 1 μm), simulated artificial seawater, and real seawater samples. The integration of the microfluidic system mitigated the interference caused by the complex seawater matrix, thereby ensuring stable and reliable microwave-based detection of microplastic concentrations. The system demonstrated high repeatability for predicting the concentration of microplastics in seawater samples. The proposed method demonstrated a recognition rate of over 96% with a tolerance of ±0.01 mg/mL, while maintaining a minimum detection limit of 48.72 ng/mL. To improve the prediction accuracy, a convolutional neural network algorithm was employed to predict microplastic concentrations based on the test results, with the predicted and actual concentrations being highly consistent. Finally, a compact and portable seawater microplastic monitoring system was developed by integrating the microwave sensor with Bluetooth and embedded systems, enabling real-time microplastic concentration monitoring in marine environments.

**Figure:**
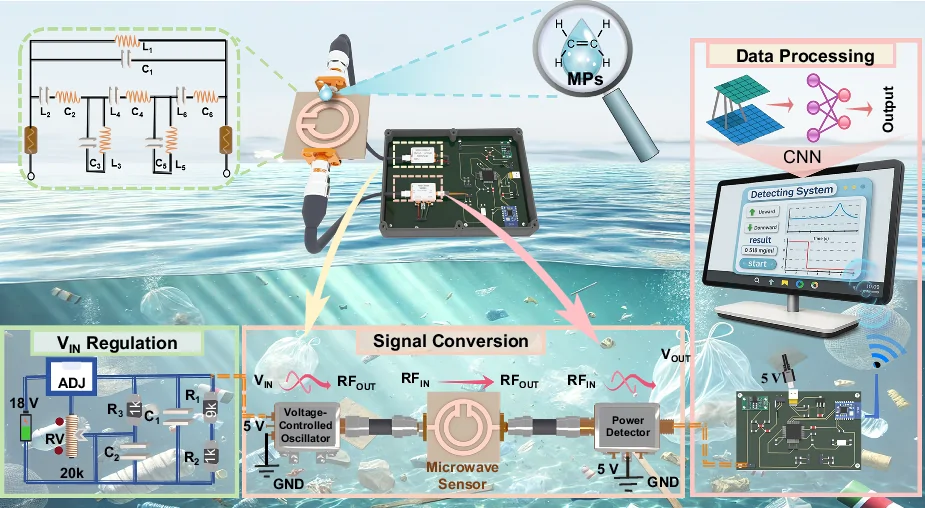

## Introduction

Microplastics, typically defined as plastic particles smaller than 5 mm in diameter^1^, have become a major global environmental challenge. The 2025 United Nations Environment Programme (UNEP) report indicates that global plastic production now exceeds 460 million tons annually, with approximately 8 million tons of plastic waste entering the ocean each year^2^. The extensive use of plastics and their low degradability have led to widespread microplastic pollution across ecosystems.

Evidence of this pollution has been widely reported. In 2018, China’s National Marine Environmental Monitoring Center documented microplastics in seawater, sea beds, and sediments^3^. More recently, concerns have extended to human health. In March 2022, scientists detected microplastics in human blood for the first time^4^. In 2024, a study at Columbia University revealed that one liter of bottled water from three top-selling brands in the U.S. contained between 110,000 and 370,000 plastic particles^5^. These microplastics can enter the food chain^6^, posing potential threats to environmental and biological health^7^. Therefore, efficient detection of microplastic concentrations in aquatic environments is critical for assessing their risks and developing effective mitigation strategies.

The global scale of marine microplastic pollution underscores the urgent need for efficient detection technologies^8^. Conventional methods, such as Fourier-transform infrared spectroscopy (FTIR)^9^, Raman spectroscopy^10^, and thermal desorption–gas chromatography–mass spectrometry (TD–GC–MS)^11^, enable high-throughput automated detection and are limited by high instrument costs, operational complexity, and stringent sample requirements (e.g., purity and particle size restrictions). In particular, spectral methods are prone to interference from particle overlap owing to their reliance on two-dimensional planar counting, while the bulky equipment restricts their use to laboratory settings. Consequently, developing cost-effective, portable, and widely applicable technologies for the rapid batch detection of microplastics remains a key challenge. Recent advances in microwave sensing technology, which exploit differences in dielectric properties, offer promising advantages^12–14^. By detecting resonant perturbations caused by the permittivity contrast between microplastics and water, quantitative analysis can be achieved with low background interference. Malyuskin first validated the feasibility of microplastic detection in soil and water using a frequency shift model^15^. Lee et al. developed a portable complementary split-ring resonator (CSRR)-based sensor for on-site microplastic concentration measurements; however, its long-term environmental stability requires further validation^16^. Barrancos et al. integrated microfluidics for the real-time detection of microplastics in water but faced challenges with simulation-experiment deviations and manufacturing precision^17^. Together, these studies demonstrate the sensitivity of microwave resonant frequency to microplastic concentrations^18^; however, practical applications remain limited by challenges in signal interpretation in complex environmental conditions.

To overcome these limitations, this study demonstrates a microwave resonant perturbation-based method for detecting microplastic pollution levels to establish a reliable real-time monitoring system for seawater microplastics^13,19^. The core innovation lies in a planar microwave sensor integrated with a microfluidic chip and a portable wireless measurement system, enabling real-time microplastic concentration monitoring^17,20^. Miniaturized packaging technology converts the sensor into a user-friendly platform, while Bluetooth connectivity enables mobile data visualization. Additionally, a convolutional neural network (CNN) algorithm optimizes the concentration prediction model, significantly improving detection accuracy and response speed^21^. This system addresses the limitations of conventional methods in terms of instrumentation cost, operational complexity, and application scenarios, while overcoming the data processing challenges of existing microwave sensors. By providing real-time dynamic data for marine microplastic monitoring, the proposed system offers a practical approach to improving monitoring efficiency, supporting marine ecosystem protection, and optimizing mitigation costs.

## Results and discussion

### Feasibility testing of microwave sensor using various solutions

**Fig. 1:**
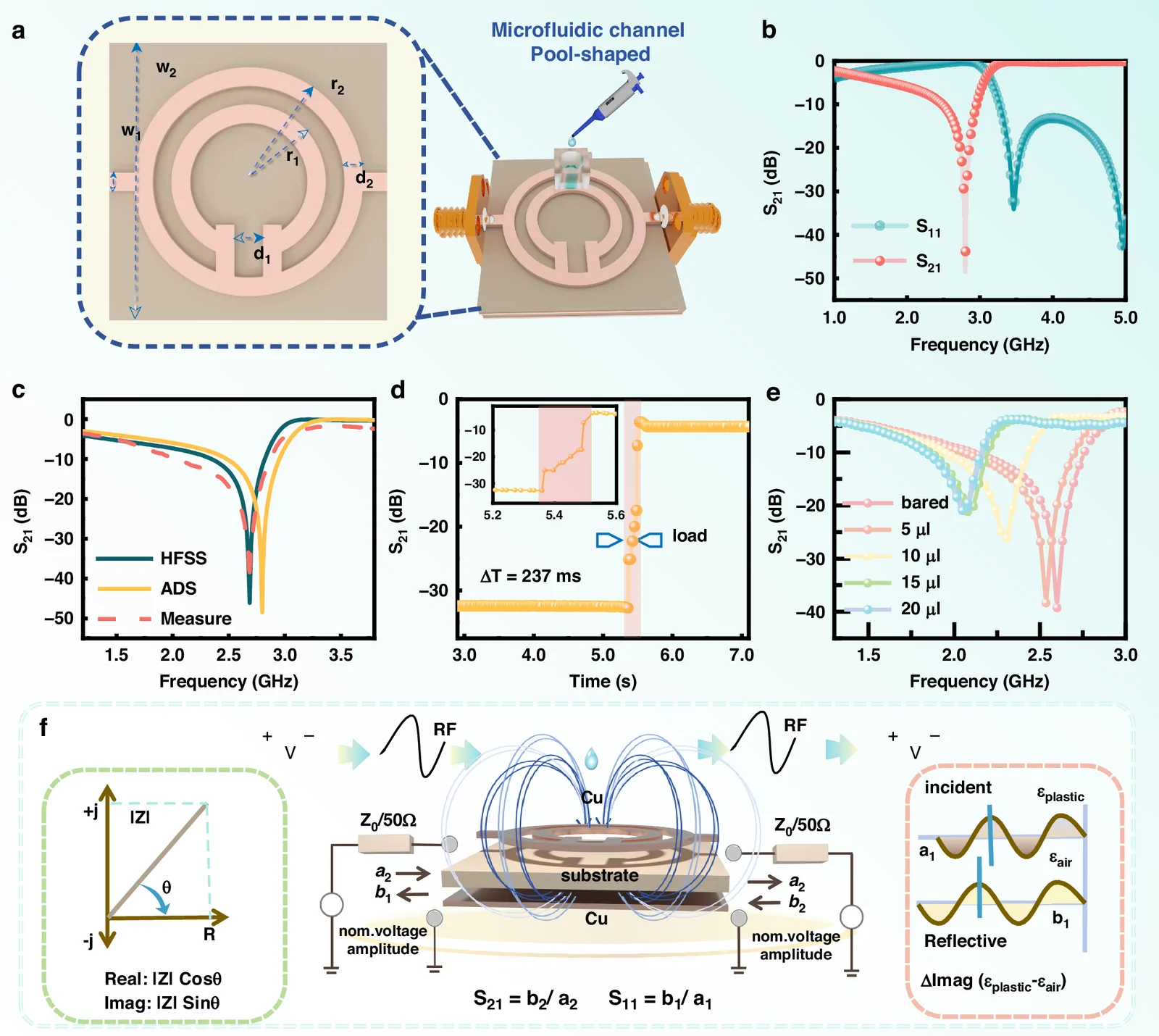
The 3D structure, simulated and measured results, and sensing mechanism of the proposed microwave sensor. **a** Schematic illustration of the resonator structure; **b** Simulated results of *S*_11_ and *S*_21_ curves by HFSS; **c** Comparison of *S*_21_ curve between simulation of HFSS, simulation of ADS and actual measurement; **d** Response time curve of the sensor after dropping the solution to be detected; **e** *S*_21_ curves of different sample dropping amounts; **f** Schematic diagram of the microwave sensor

The structural design of the microwave sensor is illustrated in Fig. 1a, with the dimensions optimized through simulations to enhance the response at the target resonant frequencies (detailed dimensions are provided in Fig. S1). Figure 1b shows the *S*_11_ and *S*_21_ response curves obtained from Ansys High Frequency Structure Simulator (HFSS), revealing a resonant frequency of approximately 2.8 GHz in the *S*_21_ response with a depth of −48.5 dB at the resonance frequency. The comparative analysis in Fig. 1c demonstrates excellent agreement between the experimental measurements and Keysight Advanced Design System (ADS) simulations for both the resonant frequency and *S*_21_ response characteristics; however, slight deviations from the HFSS results are observed. These minor discrepancies likely originated from the manufacturing tolerances of the physical device. The equivalent circuit model developed in the ADS (Fig. S2), and its corresponding parameters (Fig. S2) provide further insight into the operational mechanism of the sensor. When the electric field of the sensor interacts with the test samples, the resulting capacitance changes induce measurable resonance frequency shifts, which can be monitored in real time using a vector network analyzer (VNA).

The sensor’s working principle relies on microwave-medium interactions governed by the complex permittivity (*ε*_r_) of the medium. The real component (*ε*′) represents energy storage capacity through displacement polarization, while the imaginary component (*ε*″) characterizes energy loss from relaxation polarization processes. These fundamental parameters enable precise determination of the resonance frequency and transmission coefficient of the interdigital electrode structure, forming a theoretical basis for sensor design and optimization^22^.

1 $${\varepsilon }_{r}={\varepsilon }^{{\prime} }-j{\varepsilon }^{{\prime} {\prime} }$$

The material properties also influence the dielectric loss tangent^23^:

2 $$\tan \delta =\frac{{\varepsilon }^{{\prime} {\prime} }}{{\varepsilon }^{{\prime} }}$$

Figure 1d shows the rapid response time of the system of 237 ms. The volume-dependent effects are shown in Fig. 1e. It shows that the *S*_21_ response stabilizes when the sample volume exceeds 20 μL in the polydimethylsiloxane (PDMS) microfluidic channel. Consequently, all subsequent experiments employed a standardized 20 μL sample volume to eliminate measurement variability from volume differences. Comprehensive characterization confirmed the capability of the sensor for reliable microplastic detection across different solution matrices. The combination of a simulation-guided design, rapid response time, and standardized measurement protocol establishes a robust platform for quantitative environmental monitoring applications.

**Fig. 2:**
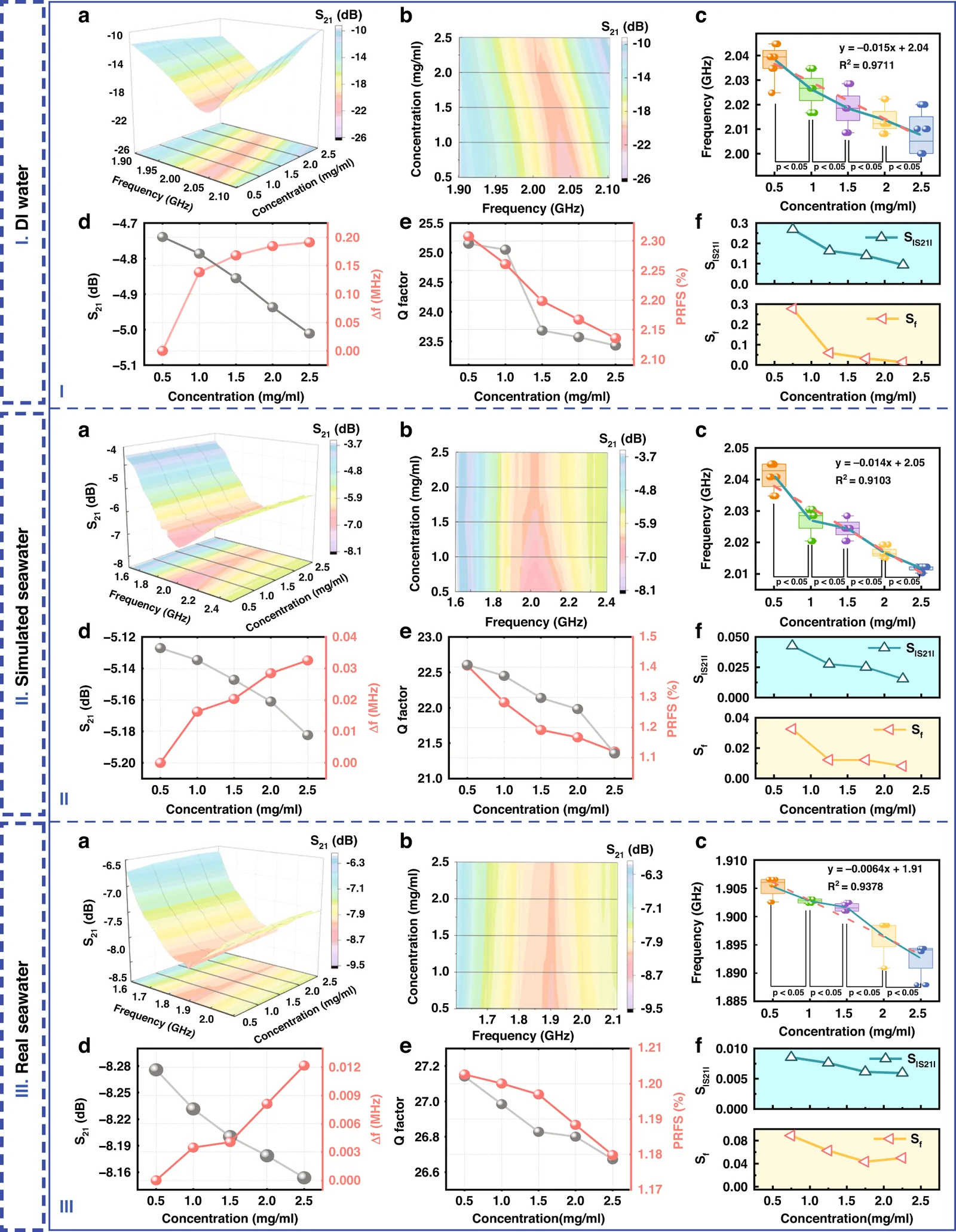
The response of the microwave sensor to different tesing samples. Test results of microplastics doped in deionized water (**I**), simulated seawater (**II**), and actual seawater (**III**). **a** 3D map of *S*_21_ results of different concentrations of microplastics. **b** *xy*-axis projection image corresponding to the 3D map. **c** Box plots of resonant frequencies of samples with different concentrations. **d** Amplitude of S21 (left) and the absolute value of frequency difference (right) at different concentrations at the difference frequency. **e** Variation of quality factor *Q* (left) and *PRFS* (right); **f** Variation of sensitivity calculated by amplitude with concentration (top) and sensitivity calculated by resonance frequency with concentration (bottom)

The experimental results demonstrate the performance of the sensor across three distinct aqueous environments, progressing from simple to complex matrices. Figure 2 presents a comprehensive evaluation of microplastic detection in deionized water (Section “Introduction”), simulated seawater (Section “Results and discussion”), and natural seawater (Section “Conclusion”), systematically validating the sensor’s capability for environmental monitoring applications.

In deionized water (Fig. 2I), the sensor exhibited distinct response patterns with varying microplastic concentrations. The 3D mapping of *S*_21_ parameters (Fig. 2Ia) and the corresponding *xy*-projection (Fig. 2Ib) revealed clear concentration-dependent signatures. The color gradients illustrate the evolution of the amplitude and resonant frequency shifts. Quantitative analysis showed a strong linear relationship between resonant frequency and concentration (*y* = −0.015*x* + 2.04, *R*² = 0.9711), as evidenced by the boxplot distributions in Fig. 2Ic. Figure 2Id illustrates the frequency dependence of the amplitude and frequency shifts across various concentrations. The amplitude exhibited a downward trend, and the variation difference was relatively uniform. The variation in frequency shifting shows an increasing trend with an increase in concentration, which leads to a large error if the concentration is predicted by simply using a linear fitting curve; therefore, the deep learning model was adopted in later research. Figure 2Ie shows a line chart of the changes in *Q* quality factor (left) and the percentage change of resonance frequency shift (*PRFS*) (right) corresponding to different concentrations. The *Q* quality factor gradually decreases with an increase in the concentration of the test sample, and the formula for calculating the *Q* quality factor is shown in Eq. 3^24^:

3 $$Q=\frac{{f}_{r}}{{f}_{r+3{dB}}-{f}_{r-3{dB}}}* 100 \% \,$$

*PRFS* is the percentage change in the resonance frequency, which gradually increases with an increase in the sample concentration. It is calculated as follows^25^:

4 $${PRFS}=\frac{{f}_{r2}-{f}_{r1}}{{f}_{r1}}* 100 \%$$

Figure 2If shows the variation trend of the amplitude variation sensitivity (top) and the resonance frequency variation sensitivity (bottom). Both sensitivities in Fig. 2If show a downward trend with the increase of sample concentration. In this study, the capacitance and inductance of the test results were also calculated, and the calculation results are shown in SI; the calculation process is shown in Eqs. 5 and 6.

5 $${Capacitance}={imag}(Y(1,1))/(2* {pi}* {frequency})$$

6 $${Inductance}={imag}(Z(1,1))/(2* {pi}* {frequency})$$

In simulated seawater (artificial sea salt solution with a salinity of 4%, Fig. 2II), the sensor maintained its detection capability despite its increased ionic strength. The 3D response patterns (Fig. 2IIa, b) and statistical distributions (Fig. 2IIc) closely mirror those observed in pure water, demonstrating the robustness of the method against controlled ionic interference. The preserved linear trends in the frequency-concentration relationships (*R*² > 0.96) and consistent behavior of the derived parameters (Fig. 2IId–f) confirmed the stability of the sensor in saline environments approaching natural seawater conditions.

The most significant validation came from testing in natural seawater (Fig. 2III), where the sensor successfully resolved microplastic concentrations amidst complex environmental interference. The characteristic 3D response patterns (Fig. 2IIIa, b) and parameter trends (Fig. 2IIIc–f) remained consistent with previous measurements, proving the environmental applicability of the method. This performance was further supported by the complementary *S*_21_ curves (Fig. S3) and phase/impedance analyzes (Fig. S4), which provide additional verification of the sensor stability under real-world conditions.

The consistent trends observed across all three matrices demonstrate the reliability of the sensor regardless of the solution complexity, with particular significance for its successful operation in natural seawater. The unified behavior of the quality factors, frequency shifts, and sensitivity metrics establishes robust quantification criteria for environmental applications. These findings represent a substantial advancement in microplastic monitoring technology that overcomes matrix effects that typically challenge conventional detection methods in marine environments. Comprehensive parameter analysis not only validates the current sensor design but also provides a foundation for developing standardized detection protocols for complex aqueous systems. Furthermore, the observed nonlinearities in concentration-response relationships highlight the necessity of employing advanced machine learning algorithms to ensure high-fidelity environmental monitoring.

### Detection of microplastics in seawater using microfluidic system

**Fig. 3:**
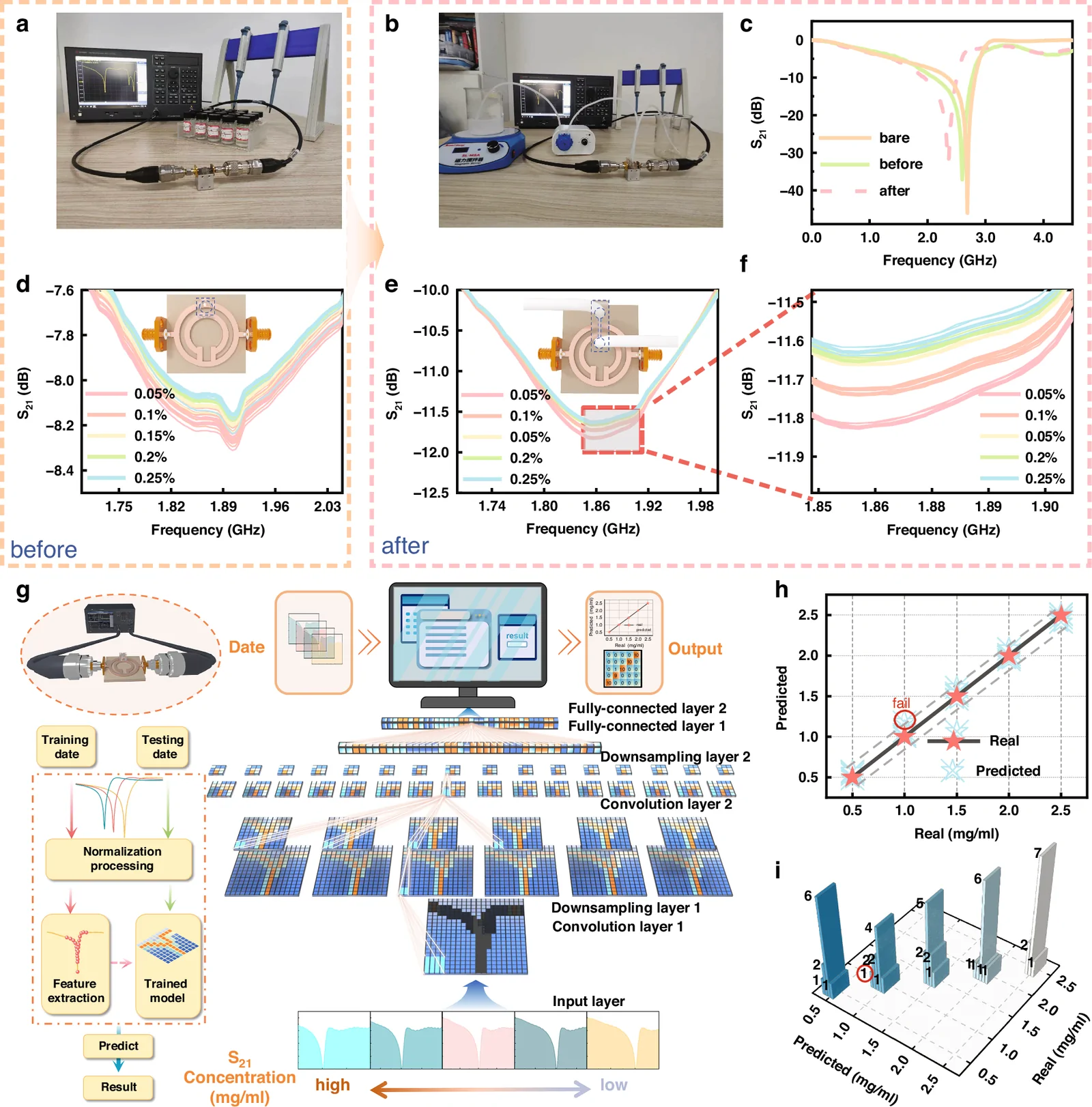
The microwave sensor was tested using a network analyzer, and combined with the Convolutional Neural Network model to predict and verify the sample data. **a** Physical images of the sensor with the pool-shaped microfluidic channel and **b** improved tubular microfluidic channel. **c** Comparison of *S*_21_ curves between the bare resonant cavity, the pool-shaped microfluidic channel (before), and the tubular microfluidic channel (after). The *S*_21_ curves corresponding to the detection of samples of different concentrations using a sensor (**d**) with a pool-shaped microfluidic channel and **e** with the improved tubular microfluidic channel. **f** Magnified view of the *S*_21_ curves. **g** Schematic of the flow of the CNN. **h** Comparison graph of the predicted values and the true values. **i** Graph showing the number of predicted values in each interval near the true values

The microfluidic detection system demonstrated significant improvements in the measurement consistency and reliability of seawater microplastic analysis. Figure 3a, b show photographs of the actual sensor integrated with two distinct microfluidic channel designs. The pool-type PDMS microfluidic device shown in Fig. 3a was designed to eliminate operational errors associated with manual sample deposition, whereas the tubular PDMS microchannel shown in Fig. 3b, coupled with an adjustable peristaltic pump, ensured a constant flow velocity through the sensor’s sensitive region.

Comparative analysis of *S*_21_ response curves in Fig. 3c revealed that microfluidic integration caused minimal frequency shifts (≤0.1 GHz) while preserving the fundamental resonance characteristics. The tubular channel induced a slightly higher frequency displacement than the pool-type design, likely because of its enhanced contact area with the sensing region. The concentration-dependent measurements in Fig. 3d, e demonstrate that both configurations could effectively distinguish microplastic concentrations, even though the tubular system showed superior reproducibility, with 10 repeated measurements per concentration. The magnified view in Fig. 3f highlights the exceptional measurement consistency achieved by the tubular system, with all the replicates forming tightly clustered curves. This high repeatability not only improved the concentration resolution but also provided superior training data for subsequent machine learning analysis. The system achieved a calculated limit of detection (LoD) of 48.72 ng/mL using Eq. (7), where *K* = 3 represents the standard deviation at 95% confidence level, *S*_0_ denotes the standard deviation, and *S* is the calibration curve slope^26^:

7 $$L{oD}=K* \frac{{S}_{0}}{S}$$

The limit of detection of the proposed system is remarkably lower than that of most reported microwave sensing methods. Although conventional techniques including FTIR, Raman spectroscopy and TD/Py-GC-MS exhibit respective strengths in laboratory qualitative analysis and source tracing, this system possesses irreplaceable advantages in portability, response speed and in-situ real-time monitoring, which makes it well suited for rapid screening of trace microplastics in marine environments. A detailed performance comparison between conventional detection methods and this work is presented in Table S3.

To improve the accuracy of concentration prediction for microplastics, machine learning was adopted in this study. Convolutional Neural Network (CNN) is a typical feedforward neural network, which generally consists of an input layer, convolutional layers, pooling layers and fully connected layers. As the core feedforward model in this work, the workflow of the CNN is illustrated in Fig. 3g. Convolutional layers can automatically extract local feature patterns from raw data, while pooling layers reduce data dimensionality and enhance the generalization capability of the model. During data preprocessing, discretization was performed on the transmission coefficients. Taking the minimum point F_0_ as the reference, five frequency points on the left side (F_-5_,F_-4_,F_-3_,F_-2_,F_-1_) and the corresponding amplitudes (M_-5_,M_-4_,M_-3_,M_-2_,M_-1_) were selected. Similarly, another five frequency points on the right side (F_+1_,F_+2_,F_+3_,F_+4_,F_+5_) and their corresponding amplitudes (M_+1_,M_+2_,M_+3_,M_+4_,M_+5_) were acquired. Combined with the quality factor Q and PRFS, a total of 24 characteristic parameters were obtained as the input features for the CNN model.

Each sample was measured in 20 replicates, half of which were used as the training set and the rest as the validation set. In total, 4800 feature values were extracted from all response data, and the detailed sample information is summarized in Table S4. The datasets cover a wide concentration range from 0.5 mg/mL to 2.5 mg/mL, which guarantees good representativeness. Benefiting from the powerful capability in handling nonlinear variations, the CNN can effectively capture inherent feature patterns. This feature extraction strategy highlights the key variation trends of response curves, which retains sufficient effective information while realizing efficient dimensionality reduction. Through convolution operations, the model can autonomously learn discriminative features and thus greatly improve the prediction accuracy.

Figure 3h shows a position comparison between the predicted and true values using the CNN. The predicted value is generally near the true value, and the prediction result shows that the correct rate reaches 98% when the allowable error range is ±0.01 mg/ml. Figure 3i shows the distribution of the predicted values near the actual values. The predicted values are all distributed near the real values in a normal distribution, which proves the accuracy and stability of the predicted results. The convergence curves of the training/validation loss functions and Root Mean Square Error (RMSE) (Fig. S5) further validate the stability and learning efficiency of the model. This combined microfluidic-CNN approach represents a significant advancement in environmental microplastic monitoring, achieving both high precision and field-deployable practicality.

### Portable microplastics detection system

#### Hardware components

**Fig. 4:**
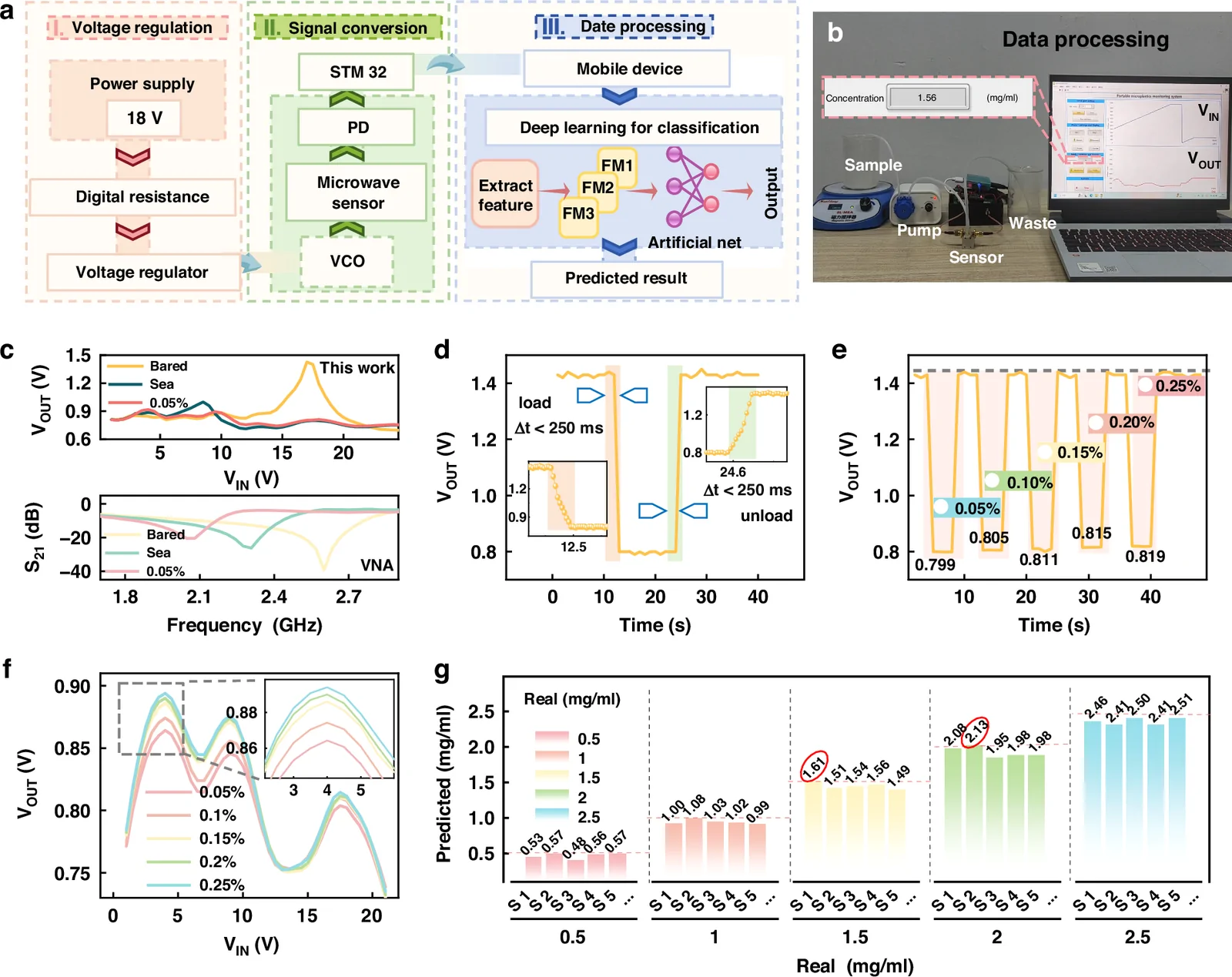
The workflow of the detection system and the physical image of the portable equipment and software interface. **a** Flowchart of the detection system; **b** Image of the detection equipment; **c** Voltage output of the bare resonant cavity, seawater, and 0.05% concentration sample with the measured results of VNAs; **d** Time response of sample loading and sample removal; **e** Output voltage gradient corresponding to the resonant frequencies of samples with different concentrations; **f** Output voltage of samples with different concentrations at sweeping frequencies; **g** Distribution of predicted values near the true values

The system architecture, illustrated in Fig. 4a, features a fully integrated hardware platform for field-deployable microplastic detection. The core detection mechanism involves precise frequency modulation using a digitally controlled voltage-tuning system. A programmable digital potentiometer (X9C503S) dynamically adjusts the input voltage (*V*_tune_) to a voltage-controlled oscillator (VCO), generating microwave signals that interact with the electromagnetic field of the sensor. The 30 V power supply, regulated through an LM315 linear regulator with adjustable output (1.25–27 V), ensures stable operation with enhanced transient response through 0.1 μF ceramic decoupling capacitors. Voltage-division monitoring via *R*_1_/*R*_2_ resistors provides real-time feedback for system calibration.

Signal processing occurs through a cascade of specialized components, and the VCO-generated microwave signal interacts with the samples in the sensing region, producing dielectric-dependent perturbations that are captured by the power detector (PD) and converted into analog voltage signals. These signals undergo analog-to-digital conversion via the STM32F103RET6 microcontroller’s 12-bit ADC before wireless transmission through a BLE 5.2 module. The physical implementation (Fig. 4b) demonstrates the system’s compact form factor, with all critical components (STM32 control board, VCO, and PD) operating on a unified 5 V supply with common grounding to eliminate potential drift-induced errors.

#### Data processing pipeline

The system employs a sophisticated dual-phase detection approach that combines hardware linearity and machine learning intelligence. As shown in Fig. S6, the VCO exhibited near-linear frequency-voltage characteristics, enabling precise frequency sweeping through digital resistance control. Figure 4c validates system performance against benchtop VNA measurements, showing excellent agreement for both blank seawater and 0.05% microplastic solutions across the 0–25 V input range. The optimized design achieved rapid response characteristics with a settling time of <250 ms for both signal acquisition and system recovery (Fig. 4d). For concentration quantification, the system operates at a fixed 2.8 GHz excitation frequency (selected for optimal sensitivity based on preliminary tests) while monitoring the PD output voltage temporal gradients during continuous microfluidic sample flow. Figure 4e shows distinct voltage trajectories corresponding to different microplastic concentrations, forming the basis for the machine learning analysis. Figure 4f shows the output voltage of the microplastic samples with different concentrations, proving that the detection system could clearly distinguish samples with different concentrations. Figure S7 illustrates the software interface. A schematic of the design circuit of the detection system is shown in Fig. S8.

This system abandons the simple method of treating the detection data as the result of a linear calibration equation, which has a large calculation error and requires absolute linearity of the detection voltage and concentration gradient. Instead, a deep learning CNN is used to predict the results. The CNN algorithm automatically extracts data features, providing strong robustness, tolerance for error changes in the input data, and mature technology to meet the requirements of real-time detection. First, the results of the output voltage obtained from samples with different concentrations were used as training sets for convolutional neural network training. Ten groups of each concentration gradient were used as training sets. Ten additional groups of samples with different concentrations were used as the validation set to predict the concentration, and the predicted values were generally close to the actual concentrations. As shown in Fig. 4g, only two groups exhibited an error of ±0.01 mg/ml relative to the actual value, resulting in 96% accuracy, which is nearly consistent with the results obtained using the VNA. Statistical analysis further revealed that the predicted values were almost normally distributed around the real values, consistent with the expected results.

## Conclusion

This paper presents a breakthrough in environmental monitoring technology through the development of a portable microwave resonance-based microplastic detection system. The integrated platform successfully addresses key challenges in field deployment by combining miniaturized hardware with advanced machine learning analytics. Systematic evaluation using 6.5 ± 1 μm polyvinyl chloride (PVC) particles demonstrated reliable detection across aqueous matrices (deionized water, artificial seawater, and natural seawater), with exceptional measurement reproducibility and prediction accuracy. The microfluidic-CNN architecture represents great progress over traditional methods by both improving the consistency of prediction and eliminating dependence on bulky instruments. Bluetooth-enabled wireless operation further enhances the practical utility for real-time environmental monitoring. These innovations establish a robust foundation for intelligent pollution management systems, offering transformative potential for large-scale microplastic surveillance in marine ecosystems. Future work will focus on expanding the detection range to include diverse polymer types and smaller particle sizes, with ongoing optimization for autonomous deployment under field conditions.

## Materials and methods

### Portable microplastic concentration detection system

**Fig. 5:**
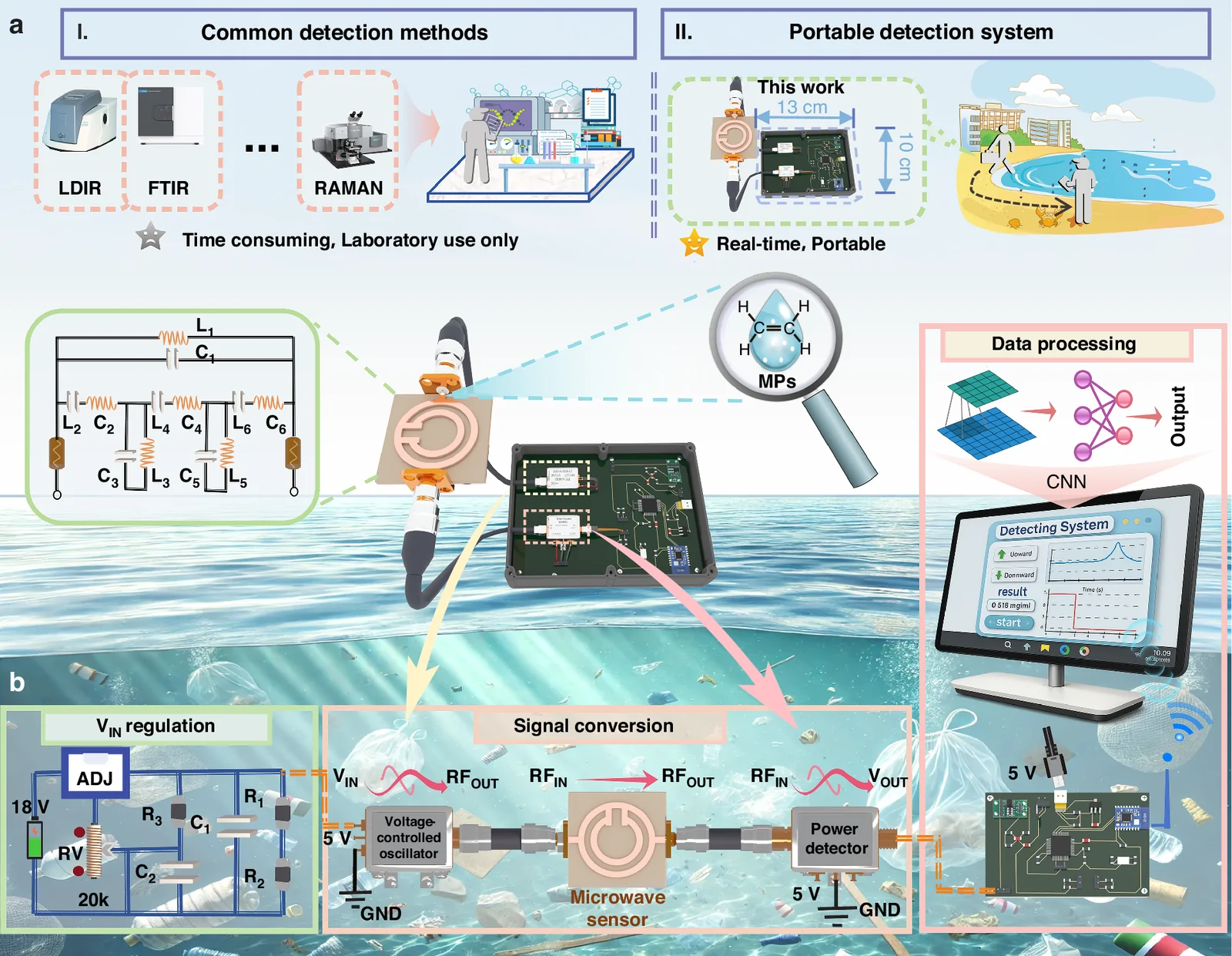
The overall architecture of a portable microplastic detection system using the proposed microwave sensor. **a** Advantages of this work compared with conventional detection methods. **b** Portable microplastics detection system includes detection mechanism and data collection through deep learning for analysis and prediction of sample concentration

A portable microplastic detection system based on microwave sensors was developed to enable real-time monitoring of microplastics in seawater^27,28^. The feasibility of the system was first validated through experiments that demonstrated the microwave sensor detection of microplastics in solution. Building on this foundation, an integrated real-time monitoring system incorporating a microfluidic chip was constructed^29,30^. Figure 5a illustrates the advantages of this method over conventional detection methods. The system utilizes an ultrasonic oscillator to disperse microplastic particles, while magnetic stirring maintains a uniform suspension. A miniature peristaltic pump delivers the sample at a constant flow rate through a microfluidic channel fabricated using 3D-printed molds and PDMS, significantly improving the detection stability and data acquisition efficiency. The experimental setup is illustrated in Fig. S9. This configuration provides a robust platform for establishing a deep learning database and training a CNN for concentration prediction^31^. When the samples flow through the sensitive region of the sensor, the dielectric interactions with the electromagnetic field generate detectable signals. These signals are transmitted via the SMA connector to a VNA for processing, with waste samples collected in a dedicated container. An advanced portable detection system featuring a PCB with an STM32F103RET6 microcontroller, VCO, PD, Bluetooth module, digital potentiometer, and voltage regulator was subsequently developed. During operation, the system’s voltage regulator receives an 18 V input, while the microcontroller adjusts the output through digital resistor control, determining the oscillator’s frequency output. The processed signals are converted to voltage, digitized, and wirelessly transmitted for real-time CNN-based analysis and are displayed through a user interface^32^. The complete 5V-powered system maintains stability through common grounding. The basic structure and detection method used in this study are illustrated in Fig. 5b.

### Sample preparation

The microplastic samples adopted in this study included polyvinyl chloride (PVC) powders of 2000 mesh, 1000 mesh and 500 mesh with corresponding particle sizes of approximately 6.5 μm, 13 μm and 25 μm, as well as 2000-mesh polyethylene (PE) and polypropylene (PP) particles. Preliminary experiments revealed that severe sedimentation and flotation of particles would occur at a concentration of 3 mg/mL, failing to form a homogeneous and stable suspension. Accordingly, five concentration gradients of 0.5, 1.0, 1.5, 2.0 and 2.5 mg/mL were selected in this work, which cover the typical concentration range of microplastics in actual seawater below 3 mg/mL. All samples were subjected to 5 min of ultrasonic oscillation to mitigate particle aggregation and improve suspension homogeneity. Deionized water, artificial seawater and natural seawater were used as experimental matrices. The artificial seawater was prepared by dissolving artificial sea salt in deionized water with a salinity of approximately 4%, consistent with that of natural seawater. Natural seawater was collected from the coastal area of Xiaomai Island in Qingdao. Given the complex composition, uneven distribution, particle aggregation, sedimentation and suspended impurities of natural seawater, a standardized pretreatment protocol tailored for the microwave sensing system was established to guarantee detection accuracy. Seawater samples were first pretreated via multi-stage membrane filtration and density separation to eliminate algae, microorganisms, plankton, organic debris and other particulate interferents. Subsequently, quantitatively configured microplastics were introduced to prepare test samples, ensuring that microplastic concentration served as the sole experimental variable and effectively mitigating non-specific responses caused by complex background interferences in seawater. As shown in Fig. S10.

Prior to detection, all samples were ultrasonically treated to achieve uniform particle dispersion. During the test, samples were kept under continuous shaking and delivered via a peristaltic pump at a constant flow rate. This approach avoids concentration deviation caused by particle sedimentation in static measurement, maintains stable and uniform sample concentration throughout the detection process, and ultimately improves the accuracy and repeatability of measurements. Finally, the key features are further extracted by CNN to further improve the accuracy of recognition. The schematic diagram of the data processing flow is shown in Fig. S11.

### Design and performance of the proposed microwave sensor

To obtain a higher sensing sensitivity, the dielectric response of the sensor at different positions was verified using an HFSS simulation. Figure S12 shows the electric field distribution of the sensor at two key resonance frequencies^33^. It can be seen that the electric field is significantly concentrated in the annular gap area in the upper part of the sensor, indicating that this is the most sensitive area. Figure S13 shows that the whole sensor is focused by the electric field at 2.8 GHz and 3.2 GHz. To determine the optimal sample placement position, the positions of three different dropped samples were simulated and evaluated^34^. Figure S13 shows the *S*_21_ response to placing samples with different dielectric constants at three different positions on the sensor using HFSS. Figure S13a–c show the *S*_21_ responses at three different positions corresponding to the sample placed directly above, to the right, and to the lower right of the sensor. The simulated resonance frequencies and amplitudes at each position are compared in the histograms shown in Fig. S13d–f. The upper part of the histogram shows the changing trend of the resonance frequency, and the lower part shows the change in the amplitude, which intuitively reflects the linear relationship between the dielectric constant of the sample and the resonance frequency and amplitude of the sensor at various positions. This indicates that the response of the sensor was most sensitive when the sample was placed in the gap in the upper half of the sensor. By contrast, when the sample was placed elsewhere, the observed changes were weak and irregular. Figure S14 shows the *S*_21_ response curves corresponding to different dielectric loss tangent values (*tanδ*) when the sample is dripped on the annular gap in the upper part of the sensor, further verifying the sensitivity of the dripping position.

Figure S15 presents the detection responses of PVC microplastics with particle sizes of 6.5 μm, 13 μm and 25 μm within the concentration range of 0.5–2.5 mg/mL, and Fig. S16 shows the corresponding parameter comparison results. All samples with different particle sizes exhibited consistent concentration-dependent response characteristics. As the microplastic concentration increased, the S_21_ resonant peak shifted towards lower frequencies, the resonant frequency decreased gradually, and the S_21_ amplitude increased synchronously. Meanwhile, the quality factor Q and relative frequency shift (PRFS) decreased continuously, and all datasets showed favorable linearity. In addition, particle size exerted a remarkable effect on the sensing performance. Smaller particles achieved more uniform dispersion, leading to a higher resonant frequency and lower scattering loss of the detection system.

The sensing responses of three typical microplastics, including PVC, PE, and PP were further compared at identical particle size and concentration, as illustrated in Figs. S17 and S18. The three types of microplastics shared similar signal variation trends, while their response intensities differed distinctly due to intrinsic dielectric properties. PVC possessed the highest dielectric constant, which contributed to the largest signal shift, optimal resonance stability and superior detection sensitivity. PE showed moderate response performance, whereas PP with the lowest dielectric constant presented the weakest signal distinguishability and the most severe scattering loss. The above results demonstrate that the proposed sensor can realize stable quantitative detection of various common microplastics. Moreover, different types of microplastics can be effectively identified by combining multiple response parameters.

Figures S19–S21 systematically investigate the effects of three typical environmental factors, namely temperature, salinity and pH, on the sensing performance. The results reveal that increases in temperature lead to synchronous rises in resonant frequency, Q factor and PRFS, while elevated salinity causes opposite variations of these resonant parameters. Both factors exert obvious interference on detection results, and corresponding compensation strategies are therefore required for practical applications. In the pH range of 4 to 10, the sensing signals show no remarkable fluctuations, demonstrating favorable resistance to pH variations. As presented in Fig. S22, a 3-week long-term stability test was conducted. The core resonant parameters of the sensor fluctuated within the normal measurement error range throughout the test, indicating excellent anti-aging and anti-corrosion capabilities. This sensor is capable of long-term and stable detection in marine environments.

### Fabrication of the microwave sensor

The microwave sensor was fabricated on a high-frequency PCB (Taconic TLX-8) substrate with a dielectric constant of 2.55, loss tangent of 0.0019, and thickness of 0.508 mm. The preparation process was as follows. First, the photoresist and diluent were mixed in a ratio of 2:1. The mixture was accurately matched with an electronic balance and a needle tube to ensure that the mixture was evenly coated on the board, to avoid incomplete development caused by the uneven distribution of the photoresist^35^. Then, it was dried in a drying oven at 90 °C for 5 min to ensure that the photoresist was completely dried before the development process. The mask was covered on the PCB with an acrylic board and exposed to UV light for 2 min. Subsequently, the exposed board was soaked in a developer solution comprised of 2 g of developing powder in 80 ml of water for approximately 2 min, and the excess parts were stripped after removal. The board was cleaned with clear water after each process, then dried, to avoid residual reagent pollution. The above steps of gluing, drying, and exposure were repeated for the reverse side of the board^36^. For the etching process, the etching powder was mixed with water in a ratio of 1:4, heated to 90 °C and continuously stirred for 15 min. Then, the PCB board was soaked in the demolding liquid (2 g demolding powder in 100 ml water), placed on a hot plate at 90 °C, and heated for approximately 10 min. Finally, the photosensitive blue oil film on the surface was peeled off using a soft brush to complete the fabrication of the sensor microstrip line. The complete preparation process is illustrated in Fig. S23.

The two types of microfluidic channels used in this study were made of PDMS, and the mold was designed using 3Dmax software and printed using a 3D printer (X1C series, Tuozhu Bambu Lab, China). The PDMS precursor consisted of liquid A and liquid B, mixed in the ratio of 1:10 to achieve suitable hardness and flexibility of the microfluidic channels^37,38^.

## Supplementary information

Supplemental material

## Data Availability

The data presented in this work are available from the lead contact upon reasonable request.
