# Supplementary material for: Portable microwave sensor system for real-time detection of microplastics in seawater
Source: Microsyst Nanoeng. 2026 Aug 24;12:302. doi: 10.1038/s41378-026-01413-y (PMC13503796; doi:10.1038/s41378-026-01413-y)
Supplement: Supplementary file 1 [file 41378_2026_1413_MOESM1_ESM.docx]

**Supplementary Information**

**Portable intelligent detection system for microplastics in seawater based on microwave sensor**

Yuxuan Hou^a^, Qingzhou Wang^a, c^, Qilong Zhang^a^, Jiaxu Liu^a^, Eun-Seong Kim^d, e,^ *, Nam-Young Kim^c^, Guanlin Li^f^, Yuanyue Li ^a^, Xiaocui Wang^b,^ *, Zhao Yao^a,^ *

^a^ *Shandong Key Laboratory of Micro-nano Packaging and System Integration, College of Electronic and Information, Qingdao University, Qingdao 266071, China*

^b^ *School of Environment and Geography, Qingdao University, Qingdao 266071, China*

^c^ *Department of Electronic Engineering, Kwangwoon University, Seoul 01897, the Republic of Korea*

^d^ *Laboratory of Molecular Pathology and Cancer Genomics, Department of Molecular Medicine and Biopharmaceutical Sciences, Graduate School of Convergence Science and Technology, Seoul National University, Seoul 08826, South Korea*

^e^ *Department of Medical and Digital Engineering, College of Engineering, Hanyang University, Seoul 04736, South Korea*

^f^ *School of Environment and Safety Engineering, Jiangsu University, Zhenjiang 212013, China*

The dimension is optimized by simulation, and a higher sensor response can be obtained at the target resonance frequency. The specific dimension of the resonator is shown in Figure S1.

**Figure S1.** Schematic diagram of the proposed microwave sensor.

**Table S1.** The specific size of the biosensor structure

| Dimension | *W*_1_ | *W*_2_ | *R*_1_ | *R*_2_ | *D*_1_ | *D*_2_ |
| --- | --- | --- | --- | --- | --- | --- |
| Unit (mm) | **1.3** | **20** | **6.7** | **4.1** | **2** | **1.3** |

Figure S2 shows the equivalent circuit model of the resonator based on ADS simulation, and the specific capacitance (C) and inductance (L) parameters of each part are shown in the table in Figure S2. When the electric field of microwave sensor is disturbed by the sample to be measured, it will cause the change of capacitance, which will lead to the corresponding shift of the resonant frequency of the device. Vector network analyzer (VNA) is used to monitor and record the response characteristics of sensors to this kind of interference in real time, thus providing direct experimental data for sensor performance evaluation. Compared with the actual sensor, the *S*_21_ and *S*_11_ curves of the simulated equivalent circuit model on the right have basically the same resonance frequency, and the slight difference may be caused by the preparation error.

**Figure S2.** The equivalent circuit diagram of the microwave sensor.

**Table S2**. The value of the equivalent circuit of the biosensor

| Dimension | *L*_1_ | *L*_2_ | *L*_3_ | *L*_4_ | *L*_5_ | *L*_6_ |
| --- | --- | --- | --- | --- | --- | --- |
| Unit | **9.1nH** | **0.88nH** | **0.933nH** | **6.9nH** | **0.737nH** | **6.5nH** |

| Dimension | *C*_1_ | *C*_2_ | *C*_3_ | *C*_4_ | *C*_5_ | *C*_6_ |
| --- | --- | --- | --- | --- | --- | --- |
| Unit | **0.01pF** | **0.785pF** | **1.4pF** | **0.26pF** | **2.04pF** | **0.24pF** |

Figure S3 shows the test results of microplastics samples doped in deionized water, simulated seawater and real seawater, respectively. The resonant frequency of all the sensors exhibits a linear shift as a function of microplastic concentration.

**Figure S3.** The measured results of different solute (a) deionized water, (b) simulated seawater, (c) real seawater.

In the Figure S4, the measured results of microplastics samples are presented, including the value of phase diagrams, capacitance and inductance.

**Figure S4.** Response of measured different solution (I) deionized water, (II) simulating seawater and (III) real seawater.

**Table S3.** Comparison between conventional detection methods and this work

| **Detection method** | **Limit of Detection** | **Portability** | **Detection Time** | **Sample Pretreatment** | **Application Scenarios** | **Reference** |
| --- | --- | --- | --- | --- | --- | --- |
| μ-FTIR | 1–10 μg/mL | Poor (6–8 kg) | 30–60 min | Complex | Laboratory | [9] |
| μ-Raman | 0.5–5 μg/mL | Poor (6–12 kg) | 20–40 min | Complex | Laboratory | [10] |
| TD/ Py-GC-MS | 0.1–1 μg/mL | Poor (80–150 kg) | 60–120 min | Complex | Laboratory | [11] |
| Microwave sensors | 50–1000 ng/mL | Moderate(2–5 kg) | <1 min | Simple | On-site | [12，13，14] |
| Microwave sensors | 48.72 ng/mL | Excellent (<1 kg) | <1 min | Simple | On-site | This work |

**Table S4.** Statistical information of samples for CNN algorithm

| **Item** | **Details** |
| --- | --- |
| Total number of samples | 200 groups |
| Solution matrix | Natural seawater |
| Detection approaches | Vector network analyzer, portable detection system (two types) |
| Microplastic concentration gradients | 0.5, 1.0, 1.5, 2.0, 2.5 mg/mL (5 levels in total) |
| Replicate tests for each condition | 20 times |
| Types of input features | Resonant frequency (11 features), S_21_ amplitude (11 features), Q factor, PRFS |
| Feature quantity per single sample | 24 |
| Total extracted features | 4800 |
| Training set | 100 groups |
| Validation set | 100 groups |

This system uses convolutional neural network algorithm to predict the sample results, and the feature extraction method used highlights the key change trend in the response curve, which not only retains sufficient information, but also effectively reduces the data dimension. With the help of convolution operation, the model can learn important feature patterns independently, thus significantly improving the accuracy of prediction. The convergence curve and REMS convergence curve of loss function of training set and verification set are shown in Figure S5, and both of them have good convergence effect.

**Figure S5.** RMSE and loss of the designed CNN algorithm.

Through practical measurement, it is verified that the voltage conversion of the voltage-controlled oscillator (VCO) is almost linear, and the power detector (PD) also has good linearity in converting frequency signals into voltage signals. The actual test data and linear fitting curve are shown in Figure S6. The system controls the output frequency of VCO by adjusting the digital resistor to control the tuning voltage at equal intervals, so as to realize the frequency sweeping function.

**Figure S6.** The output frequency of the VCO and output voltage of the PD.

Figure S7 shows the LabVIEW test interface designed by the detection system. The interface mainly consists of three parts: signal connection, adjusting input voltage, and predicting the inspection results of samples. Firstly, the serial port is selected and connected through the signal connection part, and the subsequent detection step can be carried out only after the connection is successful. Then, the system controls the input voltage by adjusting the digital resistor, and then changes the frequency of the signal output by the VCO. This part designs the function of continuously adjusting the digital resistance to adjust the frequency of the signal, that is, the frequency sweeping function. Finally, the collected data are input into the built-in connected MATLAB program of the system to realize the prediction of sample concentration.

**Figure S7.** The software UI of LabVIEW test interface.

Figure S8 is a schematic diagram of the design circuit of the detection system. The first part is the voltage adjustment module, which changes the *V*_tune_ of the VCO by adjusting the digital resistor (X9C503S) to generate the frequency of the output microwave signal. This signal is affected by the dielectric constant of the sample when it passes through the microwave sensor, and is received by the PD and converted into a voltage signal, which is monitored by STM32 microcontroller, and the analog voltage signal from the PD is converted into a digital signal. When the whole signal conversion module is completed, the collected data is sent to the computer through the Bluetooth module, and then the data is processed in real time by the computer.

**Figure S8.** schematic diagram of the PCB measurement system.

Figure S9 shows the testing process of the whole microfluidic system. When the sample solution flows through the sensitive area of the microwave sensor, its dielectric constant interacts with the electromagnetic field of the sensor, and the signal is transmitted to the vector network analyzer for display through the SMA connector, and finally the sample is discharged into the waste liquid bottle.

**Figure S9.** The schematic diagram of the microplastics detection system with the microfluidic device.

Figure S10 illustrates the standardized pretreatment workflow for seawater samples. To eliminate interferences from algae, plankton, organic debris and suspended particles in natural seawater, a multi-stage purification strategy combining membrane filtration and density separation was adopted. Large-sized contaminants including plankton, algal aggregates and coarse suspended solids were first removed using a 10 μm filter membrane. Afterwards, static density separation was performed based on the density difference between microplastics and biological particles. This approach effectively separates microplastics from similarly sized microbial and algal impurities, greatly reducing non-specific interference caused by complex water matrices and preparing purified and homogeneous samples for subsequent detection.

**Figure S10.** Schematic diagram of sample processing flow.

Figure S11 presents the data processing flowchart of the convolutional neural network (CNN) based multi-feature intelligent recognition method. Only trace fine impurities remain in the pretreated seawater, which may still cause slight background interference. Herein, multiple resonant response characteristics including S-parameters, quality factor Q and relative frequency shift were extracted to establish a joint discrimination model. Distinct differences in dielectric properties between microplastics (relative permittivity: 2–5) and biological impurities (relative permittivity: 60–80) lead to divergent resonant shift, signal attenuation and waveform variation patterns. The CNN model can autonomously learn the unique radio frequency response features of microplastics, distinguish disordered signals induced by biological contaminants, and extract valid detection information from massive datasets. It enables intelligent differentiation between target microplastics and residual interferents, and remarkably improves the specificity and accuracy of microplastic detection in complex marine environments.

**Figure S11.** Schematic diagram of data processing process of CNN.

In order to obtain higher sensing sensitivity, the dielectric response of the sensor at different positions is verified by HFSS simulation. Figure S12 shows the results of the electric field distribution of the sensor at two key resonance frequencies. It can be seen that the electric field is significantly concentrated in the annular gap area in the upper part of the sensor, indicating that this is the most sensitive area. Figure S12 shows that the whole sensor is focused by the electric field at 2.8 GHz and 3.2 GHz. In order to determine the optimal sample placement position, the positions of three different dropping samples were simulated and evaluated.

**Figure S12.** The simulated electric field distribution of the microwave sensor at 3.5 GHz and 2.8 GHz.

Figure S13 presents the simulated *S*_21_ response from HFSS when samples of varying dielectric constants are placed at three different sensor locations. Figure S13(a)–(c) show the *S*_21_ results for samples positioned above, to the right, and to the lower right of the sensor, respectively. The corresponding resonance frequencies and amplitudes are summarized in histograms in Figure S13(d)–(f), where the upper section illustrates the shift in resonance frequency and the lower section shows the amplitude variation. These plots clearly demonstrate a linear relationship between the sample's dielectric constant and both the resonance frequency and amplitude at each position. It indicates that the sensor exhibits the highest sensitivity when the sample is placed in the gap on the upper half of the sensor, whereas responses at other locations are weaker and less consistent.

**Figure S13.** The simulated results of *S*_21_ response on different position of the sensor (a) the upper side; (b) the right side; (c) the lower side; The statistical histogram of resonance frequency and amplitude corresponding to different place (d) on that upper side; (e) the right side; (f) the lower side.

Figure S14 shows the *S*_21_ response curves corresponding to different dielectric loss tangent values (tanδ) when the sample is dripped on the annular gap in the upper part of the sensor, which further verifies the sensitivity of the dripping position.

**Figure S14. T**he simulation results correspond to different tanδ.

Figure S15 depicts the resonant response characteristics of the sensor towards PVC microplastics with particle sizes of 6.5 μm, 13 μm and 25 μm under the concentration gradient ranging from 0.5 to 2.5 mg/mL. Figure S15(a) shows the *S*_21_ curves of 6.5 μm PVC samples. As the PVC concentration increases, the resonant peak shifts monotonically towards lower frequencies and the resonant depth decreases slightly, exhibiting a regular concentration-dependent variation. Figure S15(b) presents the corresponding variations of resonant frequency and amplitude. The quantitative results reveal that the resonant frequency decreases continuously while the *S*_21_ amplitude rises slightly with increasing concentration. As shown in Figure S15(c), both the quality factor Q and relative frequency shift (PRFS) decline gradually, and all data exhibit excellent linearity. Figures S15(d)–(f) show the detection results of 13 μm PVC microplastics. The *S*_21_ curves also shift toward lower frequencies with increasing concentration. Compared with the 6.5 μm samples, the overall resonant frequency is slightly lower and the variation of resonant depth becomes milder. Figures S15(g)–(i) present the results of 25 μm PVC samples, which exhibit the same low-frequency shift trend of resonant peaks as the above two groups.

**Figure S15.** Microwave sensing responses of PVC samples with different particle sizes. (a) *S*_21_ curves, (b) variations of resonant frequency and amplitude, (c) variations of Q factor and PRFS for 6.5 μm PVC; (d) *S*_21_ curves, (e) variations of resonant frequency and amplitude, (f) variations of Q factor and PRFS for 13 μm PVC; (g) *S*_21_ curves, (h) variations of resonant frequency and amplitude, (i) variations of Q factor and PRFS for 25 μm PVC.

Figure S16(a) compares the variations of resonant frequency and amplitude among PVC samples with three different particle sizes. The response curves show distinct distribution characteristics. The 6.5 μm samples are located in the relatively high-frequency and low-loss region, while the 25 μm samples fall into the low-frequency and high-loss region. With the increase of concentration, all three groups exhibit a low-frequency shift of resonant frequency and a rise in *S*_21_ amplitude at the resonant peak. Such distribution differences are mainly attributed to the uniform dispersion of fine particles, which exert a more remarkable modulation effect on the overall dielectric constant of the solution and thus yield a higher resonant frequency.

Figure S16(b) presents the variations of Q factor for PVC samples of different particle sizes. The Q values of all samples decrease monotonically with increasing concentration. The 6.5 μm samples maintain the highest Q factor with the slightest attenuation, whereas the 25 μm samples show the lowest Q factor and the most severe attenuation. This result indicates that the increase in particle size significantly enhances the scattering loss of the resonant system and reduces the sharpness of resonant peaks.

Figure S16(c) illustrates the changes in PRFS for the three groups of samples. PRFS declines gradually as the concentration rises, and the initial sensitivity increases with particle size. The 25 μm samples always possess the highest PRFS, which can be explained by the stronger perturbation effect of large particles on the microwave field.

**Figure S16.** Comparison of microwave sensing responses of PVC samples with three different particle sizes. (a) Variations of resonant frequency and amplitude; (b) Variation of Q factor; (c) Variation of PRFS.

Figures S17(a)–(c) illustrate the resonant response characteristics of PVC microplastics at concentrations ranging from 0.5 to 2.5 mg/mL. Figure S17(a) displays the *S*_21_ curves of PVC at different concentrations. As the PVC concentration increases, the resonant peak shifts monotonically toward lower frequencies, accompanied by a slight reduction in resonant depth. Figure S17(b) depicts the variations of resonant frequency and amplitude, showing that the resonant frequency decreases linearly while the *S*_21_ amplitude rises steadily with increasing concentration. Figure S17(c) presents the trends of Q factor and PRFS, which reflect the energy loss and relative frequency shift of the resonant system. Both the Q factor and PRFS decrease monotonically and linearly as the concentration elevates. Figures S17(d)–(f) show the detection results of PE microplastics under the same concentration gradient. Compared with PVC, PE exhibits a smaller shift of resonant peaks and milder overall variations. Its Q factor is slightly higher, and the PRFS declines more gently. Figures S17(g)–(i) correspond to the results of PP microplastics. PP shares the same concentration-dependent variation trends as the other two materials, yet it delivers the weakest overall signal shift among the three types of microplastics.

**Figure S17.** Microwave sensing responses of different microplastic samples. (a) *S*_21_ curves, (b) variations of resonant frequency and amplitude, (c) variations of Q factor and PRFS for PVC; (d) *S*_21_ curves, (e) variations of resonant frequency and amplitude, (f) variations of Q factor and PRFS for PE; (g) *S*_21_ curves, (h) variations of resonant frequency and amplitude, (i) variations of Q factor and PRFS for PP.

Figure S18 compares the sensing performances of 6.5 μm PVC, PE and PP microplastics across the concentration range of 0.5–2.5 mg/mL. Figure S18(a) shows the combined variations of resonant frequency and *S*_21_ amplitude. All three materials exhibit identical concentration-dependent trends and present distinct distribution patterns, which are primarily caused by their differences in relative permittivity. PVC has the highest relative permittivity and exerts the most prominent modulation on the dielectric properties of the solution. By contrast, PP possesses the lowest resonant frequency and the largest absolute *S*_21_ amplitude, indicating that it introduces more severe scattering and absorption losses, which is closely associated with its surface properties and dispersion state.

Figure S18(b) displays the variation of Q factor. The Q values of all three microplastics decrease monotonically with increasing concentration, with obvious differences in initial values and attenuation rates. PVC maintains the highest Q factor and the slightest attenuation, demonstrating the least adverse impact on sensor resonance and the optimal resonance stability.

Figure S18(c) presents the PRFS variation curves. PRFS declines monotonically for all samples. PVC has the highest initial PRFS with a moderate decreasing rate, revealing the best frequency response sensitivity.

**Figure S18.** Comparison of responses among three types of samples. (a) Variations of resonant frequency and amplitude; (b) Variation of Q factor; (c) Variation of PRFS.

Figure S19 illustrates the resonant responses of the sensor at different temperatures. Figure S19(a) shows the *S*_21_ curves under varied temperatures. As temperature rises, the resonant peak shifts monotonically towards higher frequencies with a regular profile, and the resonant depth gradually decreases. Figure S19(b) depicts the temperature-dependent variations of resonant frequency and amplitude. The resonant frequency increases continuously, while the absolute value of *S*_21_ amplitude declines with rising temperature, suggesting that elevated temperature reduces resonant loss and strengthens the response signal.

Figure S19(c) presents the trends of Q factor and PRFS. Both parameters increase significantly and linearly with temperature, proving that higher temperature can sharpen resonant peaks and improve the frequency resolution of the sensor, which is consistent with the narrowed resonant peaks observed in Figure S19(a). The results demonstrate that temperature is a critical environmental factor affecting the detection stability of the microwave sensor. Temperature compensation or control is therefore required in practical microplastic detection to eliminate its interference on quantitative analysis.

**Figure S19.** Microwave responses of samples at different temperatures. (a) *S*_21_ curves; (b) Variations of resonant frequency and amplitude; (c) Variations of Q factor and PRFS.

Figure S20. compares the resonant responses of the sensor under different salinity conditions. Figure S20(a) presents the corresponding *S*_21_ curves. As the salinity increases from 2% to 4%, the resonant peak shifts monotonically to lower frequencies and the resonant depth decreases gradually. Although the overall curve shape remains regular, the sharpness of the resonant peak deteriorates.

Figure S20(b) shows the salinity-dependent variations of resonant frequency and amplitude. The resonant frequency decreases continuously, while the *S*_21_ amplitude rises with increasing salinity. This phenomenon indicates that higher salinity aggravates resonant loss and dissipates signal energy.

Figure S20(c) displays the changes in Q factor and PRFS. Both parameters decrease evidently and linearly with rising salinity, demonstrating that high salinity introduces additional conductive loss, broadens resonant peaks and reduces the frequency resolution of the sensor, which is in good agreement with the peak broadening observed in Figure S20(a). The results reveal that salinity exerts a notable interference on microplastic detection. Accordingly, it is necessary to develop salinity compensation algorithms in further studies to enhance the detection stability and quantitative accuracy of the sensor in complex seawater matrices.

**Figure S20.** Microwave responses of samples with different salinity levels. (a) *S*_21_ curves; (b) Variations of resonant frequency and amplitude; (c) Variations of Q factor and PRFS.

Figure S21 shows the resonant response characteristics of the sensor under different pH conditions. Figure S21(a) presents the *S*_21_ curves across a wide pH range of 4 to 10. The curves are highly overlapped, with no obvious changes in the position, shape or depth of resonant peaks, and no monotonic shift trend is observed with pH variation.

Figure S21(b) illustrates the variations of resonant frequency and amplitude against pH. The resonant frequency only fluctuates slightly within a narrow range, with a maximum shift below 0.3 MHz. The *S*_21_ amplitude also shows minor fluctuations without a distinct pH-dependent tendency.

Figure S21(c) depicts the changes of Q factor and PRFS. Both parameters remain stable within a limited range and exhibit no obvious rising or falling trends, further proving that pH variation has negligible impacts on the energy loss and frequency shift of the resonant system. This can be explained by the fact that pH changes in seawater mainly affect the ionic hydrolysis equilibrium and acid-base buffer system, and exert far weaker effects on the overall dielectric constant and electrical conductivity of the solution than temperature and salinity. Within the tested range, pH variation causes no significant interference to the detection results.

**Figure S21.** Microwave responses of samples with different pH values. (a) *S*_21_ curves; (b) Variations of resonant frequency and amplitude; (c) Variations of Q factor and PRFS.

To verify the long-term operational reliability of the sensor in practical marine environments, a three-week continuous stability test was conducted, as presented in Figure S22. The experiments were carried out in standard seawater at 20 °C, with a salinity of 4% and pH of 8. Both the bare sensor and the solution containing 0.5 mg/mL PVC microplastics were tested. The core resonant parameters were compared between the initial measurement and the re-test performed three weeks later.

The results demonstrate that the key performance indicators of the sensor did not degrade remarkably after three weeks of service and environmental exposure. For the bare sensor, only an extremely slight shift in resonant frequency and minor fluctuations of *S*_21_ amplitude were observed, and all parameter variations fell within the normal measurement error range of microwave sensors. Slight deviations were detected after the microplastic solution was introduced, which was probably caused by inevitable residual substances on the sensor surface after cleaning. In this case, the dielectric property of the sample became the dominant factor affecting sensor responses.

It is confirmed that the sensor possesses excellent corrosion resistance and anti-aging capability, and its core detection performance is barely affected by long-term service and environmental exposure.

**Figure S22.** The results of long-term stability test.

The fabrication process is as follows: First, photoresist and diluent are mixed at a 2:1 ratio and uniformly coated on the substrate to ensure even development. The board is then dried at 90 °C for 5 minutes. A mask is applied using an acrylic plate, and the board is exposed under UV light for two minutes. The exposed board is developed in a solution of 2 g developer powder in 80 ml water for two minutes, followed by rinsing and drying. The same steps—coating, drying, and exposure—are repeated for the reverse side. Etching is performed in a 1:4 etching powder-to-water solution at 90 °C under continuous stirring for about 15 minutes. Finally, the board is immersed in a stripping solution (2 g stripping powder in 100 ml water) at 90 °C for 10 minutes, and the remaining photoresist is removed with a soft brush. The complete process is illustrated in Figure S23.

**Figure S23.** The preparation flow chart of the microwave sensor.
